# Increased Short-Term Beat-To-Beat Variability of QT Interval in Patients with Acromegaly

**DOI:** 10.1371/journal.pone.0125639

**Published:** 2015-04-27

**Authors:** Andrea Orosz, Éva Csajbók, Csilla Czékus, Henriette Gavallér, Sándor Magony, Zsuzsanna Valkusz, Tamás T. Várkonyi, Attila Nemes, István Baczkó, Tamás Forster, Tibor Wittmann, Julius Gy. Papp, András Varró, Csaba Lengyel

**Affiliations:** 1 Department of Pharmacology and Pharmacotherapy, Faculty of Medicine, University of Szeged, Szeged, Hungary; 2 1st Department of Internal Medicine, Faculty of Medicine, University of Szeged, Szeged, Hungary; 3 2nd Department of Internal Medicine and Cardiology Center, Faculty of Medicine, University of Szeged, Szeged, Hungary; 4 MTA-SZTE Research Group of Cardiovascular Pharmacology, Hungarian Academy of Sciences, Szeged, Hungary; University of Adelaide, AUSTRALIA

## Abstract

Cardiovascular diseases, including ventricular arrhythmias are responsible for increased mortality in patients with acromegaly. Acromegaly may cause repolarization abnormalities such as QT prolongation and impairment of repolarization reserve enhancing liability to arrhythmia. The aim of this study was to determine the short-term beat-to-beat QT variability in patients with acromegaly. Thirty acromegalic patients (23 women and 7 men, mean age±SD: 55.7±10.4 years) were compared with age- and sex-matched volunteers (mean age 51.3±7.6 years). Cardiac repolarization parameters including frequency corrected QT interval, PQ and QRS intervals, duration of terminal part of T waves (T_peak_-T_end_) and short-term variability of QT interval were evaluated. All acromegalic patients and controls underwent transthoracic echocardiographic examination. Autonomic function was assessed by means of five standard cardiovascular reflex tests. Comparison of the two groups revealed no significant differences in the conventional ECG parameters of repolarization (QT: 401.1±30.6 ms *vs* 389.3±16.5 ms, corrected QT interval: 430.1±18.6 ms *vs* 425.6±17.3 ms, QT dispersion: 38.2±13.2 ms *vs* 36.6±10.2 ms; acromegaly *vs* control, respectively). However, short-term beat-to-beat QT variability was significantly increased in acromegalic patients (4.23±1.03 ms *vs* 3.02±0.80, *P*<0.0001). There were significant differences between the two groups in the echocardiographic dimensions (left ventricular end diastolic diameter: 52.6±5.4 mm *vs* 48.0±3.9 mm, left ventricular end systolic diameter: 32.3±5.2 mm *vs* 29.1±4.4 mm, interventricular septum: 11.1±2.2 mm *vs* 8.8±0.7 mm, posterior wall of left ventricle: 10.8±1.4 mm *vs* 8.9±0.7 mm, *P*<0.05, respectively). Short-term beat-to-beat QT variability was elevated in patients with acromegaly in spite of unchanged conventional parameters of ventricular repolarization. This enhanced temporal QT variability may be an early indicator of increased liability to arrhythmia.

## Introduction

Hypertension, left ventricular hypertrophy, asymmetric septal hypertrophy, cardiomyopathy, and congestive heart failure are well-known cardiovascular complications of acromegaly caused by pituitary tumors [1]. Excessive secretion of growth hormone and IGF-1 can result in major structural and functional changes in cardiac system and arrhythmias, hypertension, and valvular heart disease are present in up to 60% of patients by the time of the diagnosis of acromegaly [1]. Clinical data suggest that a specific cardiomyopathy develops in acromegaly associated with life-threatening dysrhythmias [2]. Complex morphological and functional remodeling may be partially reversed by effective control of growth hormone and IGF-1 concentrations [2]. Moreover, acromegaly can also be associated with cardiovascular diseases contributing to increased mortality among patients [1,2]. Effective control of acromegaly with pegvisomant, a GH receptor antagonist, led to a significant improvement of Framingham risk score, and reduced the likelihood for development of coronary heart diseases, too [3].

Electrocardiographic studies also indicated cardiac rhythm abnormalities in patients with acromegaly [2,4,5]. Dysrhythmias, atrioventricular conduction delay and sick sinus syndrome were reported in sudden death in acromegalic heart disease [6]. Rodrigues *et al*. [7] found arrhythmias in 41% of 34 patients with acromegaly, thirteen patients had frequent ventricular extrasystoles and there were long periods of asymptomatic ventricular bigeminy in one patient. Both prevalence and severity of ventricular arrhythmia were significantly higher in acromegalic patients compared to controls, and the frequency of ventricular premature complexes increased with duration of acromegaly [8]. Higher incidence of late potential positivity, QT interval prolongation and higher QT dispersion in acromegaly patients might explain the increased susceptibility to sudden cardiac deaths from ventricular tachyarrhythmias [9]. Herrmann *et al*. [10] detected late potentials, a predictor of ventricular dysrhythmias in a signal-averaged electrocardiogram, in 56% of patients with active acromegaly (n = 16) and 6% of well-controlled patients (n = 32) and speculated that late potentials might indicate myocardial remodeling in acromegaly. In another study, the occurrence of late potentials were 22.9% in acromegalic *vs* 2.9% in control patients (*P*<0.001; n = 70 in both groups) and a significant association with premature ventricular complexes were seen by means of 24-h Holter ECG recording [11]. Maffei *et al*. [11] also described that one case of sudden cardiac death occurred during the observation period, and this acromegalic patient had late potentials, left ventricular hypertrophy, Lown 4 premature ventricular complexes, and non-sustained ventricular tachycardia.

The identification of patients with risk for serious ventricular arrhythmia and sudden cardiac death could be important during the diagnosis and treatment of acromegaly. Fatti *et al*. [12] described that octreotide, a somatostatin analogue, could improve abnormally prolonged QT interval in acromegalic patients. Treatment with GH receptor antagonist Pegvisomant for 6-month and 18-month (long-term) also improved rhythm abnormalities in 13 patients suffering from acromegaly [13]. However, QT interval prolongation alone cannot reliably predict the development of ventricular arrhythmias including the chaotic ventricular tachycardia, Torsades de Pointes (TdP), since cardiac repolarization reserve may be reduced even without significant changes in the duration of cardiac repolarization [14]. The short-term variability of the duration of repolarization (STV_QT_) [15] might be a better parameter to predict serious ventricular arrhythmias and sudden cardiac death, as it has been suggested by both animal experimental work [16–19] and recent clinical studies [20–23]. On the basis of these observations, Varkevisser *et al*. [24] suggested that beat-to-beat STV_QT_ could be superior to QT interval prolongation in identifying patient populations at risk for ventricular arrhythmias and might be able to accurately predict individual risk. The aim of the present study was to determine beat-to-beat QT variability in patients with acromegaly.

## Methods

### Patient Population

Patients with acromegaly who are followed at the 1^st^ Department of Internal Medicine in Szeged, Hungary, were eligible for this study. Patients were excluded if they had excessive (>5%) ectopic atrial or ventricular beats, were in a rhythm other than normal sinus, had repolarization abnormalities (i.e. early repolarization pattern, T wave inversion and complete left bundle branch block or right bundle branch block), had a permanent pacemaker or any other disorders such as serious retinopathy, symptomatic cardiac and pulmonary disease, acute metabolic disease, had excessive noise on the electrocardiographic signal that precluded analysis of the ECG waveform, were on any medication likely to affect the investigated ECG parameters or consumed significant amount of food within 3 hours or drank alcohol, coffee or smoked within 10 hours.

We studied 30 acromegalic patients, 7 males and 23 females with the age of 55.7 ± 10.4 years (all values presented are mean ± SD). A total of 30 age- and sex-matched volunteers (mean age 51.3 ± 7.6 years) without a history or evidence of heart disease were enrolled in the study as controls. All of the control individuals and acromegaly patients were of Caucasian origin. Acromegalic patient group was also divided to subgroups on the basis of medical examinations and serum diagnostic tests performed (hGH rhythm, IGF-1 level, HbA1c concentration, oral glucose tolerance test). Active acromegalic subgroup included acromegalic patients before hypophysectomy or with remnant hormonally active tumor after hypophysectomy (n = 14), as well as treated acromegalic patients with high serum IGF-1 levels in spite of long-acting somatostatin analogue octreotide or lanreotide therapy received (n = 3). Inactive acromegalic subgroup included acromegalic patients after successful hypophysectomy (n = 6) and treated acromegalic patients with an age-sex-appropriate normal IGF-1 and/or random GH < 1 ng ml^-1^ and/or nadir GH after OGTT < 0.4 ng ml^-1^ during bromocriptine, pegvisomant, or long-acting somatostatin analogue octreotide treatments (n = 7). There was no significant age difference between the active and inactive patients (56.4 ± 11.5 vs 54.8 ± 9.2 years, respectively; *P* = 0.69). In acromegaly group, there were 18 hypertensive patients receiving therapy (for details see S1 Table) and 12 normotensive subjects, whereas volunteers in control group did not receive antihypertensive treatment.

The studies described here were carried out in accordance with the Declaration of Helsinki (2000) of the World Medical Association and were approved by the Scientific and Research Ethical Committee of the Medical Research Council at the Hungarian Ministry of Health (ETT-TUKEB), under ethical approval No. 4987-0/2010-1018EKU (338/PI/010). All subjects have given written informed consent of the study.

### Data Collection and Analysis

12-lead electrocardiograms were continuously recorded for 5 min at rest, in the supine position to obtain signals with the least amount of motion artefact. In all leads the ECG signals were digitized at 2000 Hz sampling rate with a multichannel data acquisition system (Cardiosys-H1 software, Experimetria Ltd, Budapest, Hungary) connected to a personal computer and stored for later off-line analysis.

Out of the repolarization parameters we analyzed the frequency corrected QT interval (QTc) using Bazett’s (QTc = QT/√RR), Fridericia (QTc = QT/[RR/1000]1/3), Framingham (QTc = QT + [0.154 * {1000-RR}]) and the Hodges formulas (QTc = QT + 1.75 * [60 000/RR-60]), the QT dispersion (QTd), the PQ and QRS intervals, the duration of terminal part of T waves (T_peak_-T_end_) and the short-term variability of QT interval (STV_QT_).

The RR and QT intervals and duration of the T wave from the peak to the end (T_peak_-T_end_) intervals were measured automatically in 30 consecutive beats (minimum number of intervals needed for variability measurements), were checked by the same expert investigator of the team for all ECGs and manually corrected if needed and were calculated as the average of 30 beats. QTc interval duration was defined as the mean duration of all QTc intervals measured. The PQ and QRS intervals were measured as the average of 15 consecutive beats. All measurements were carried out using limb lead II and in case of excessive noise in limb lead II and lead V5.

To characterize the temporal instability of beat-to-beat repolarization, Poincaré plots of the QT intervals were constructed, where each QT value is plotted against its former value. STV_QT_ was calculated using the following formula: STV_QT_ = ∑|QT_n+1_-QT_n_|/(30x√2), where QT represents the duration of the QT interval. This calculation defines the STV as the mean distance of points perpendicular to the line of identity in the Poincaré plot and relies on previous mathematical analysis (25).

All acromegalic patients and controls also underwent transthoracic echocardiographic examination performed by the single observer blinded to subject data for all participants. Two-dimensional echocardiographic images were obtained by Toshiba Powervision 8000 echocardiography equipment, in a number of cross-sectional planes using standard transducer positions to determine standard morphological and functional parameters.

Autonomic function was assessed by means of five standard cardiovascular reflex tests: the heart rate (HR) responses to deep breathing and to standing up (30/15 ratio), the Valsalva maneuver, the systolic blood pressure response to standing up, and the diastolic pressure change during a sustained handgrip. A score was created to express the severity of autonomic neuropathy (AN), based on the results of the five tests (normal: 0, borderline: 1, abnormal: 2). The total score was in the interval of 0 to 10.

Fasting venous blood samples were obtained from each patient and controls for the determination of serum glucose, blood urea nitrogen, creatinine, sodium and potassium levels. GH and IGF-1 were measured by chemiluminescent immunoassay (IMMULITE 1000 Immunoassay System, Siemens. GH measurement comparator: Recombinant 98/574; detection limit: 0.01 ng ml^-1^; intra-assay coefficients of variation: 6.0%; interassay coefficients of variation: 6.2%. IGF-1 measurement comparator: WHO IRP 87/517; detection limit: 20.0 ng ml^-1^; intra-assay coefficients of variation: 5.0%; interassay coefficients of variation: 9.0%).

### Statistical Analysis

All data are expressed as mean±SD. Comparisons between acromegalic patients and controls for the study variables were done using the unpaired Student’s *t* test for normally distributed parameters, nonparametric Mann-Whitney U test for non-normal distributions, and linear regression for revealing correlations. The statistical analyses were performed using the SPSS 16.0 software package. Statistical significance was accepted at the *P*<0.05 level.

## Results

### Clinical data of acromegalic patients and control subjects

In 30 acromegalic patients studied, body weight and mean body mass index (BMI) were significantly higher (*P*<0.001 for both parameters) than those in age- and sex-matched volunteers (Table 1). Mean systolic blood pressure did not differ significantly between control subjects and acromegalic patients receiving standard care and treatment, however, acromegalic patients had higher diastolic blood pressure (*P*<0.05). The incidence of high blood pressure was 7/30 in control and 13/30 in acromegaly groups during the actual measurements. Average serum glucose and HbA1c values were also similar in both groups; incidences of diabetes were 0/30 and 1/30 in control and acromegaly groups, respectively. Incidence of impaired glucose tolerance was 0/30 in control and 4/30 in acromegalic subjects. Significant differences were seen in serum hGH (*P* = 0.0028) and IGF-1 (*P* = 0.0013) levels between acromegalic and control groups. There was no significant difference in nadir value of hGH during oral glucose tolerance test (OGTT) between active (3.40 ± 2.10 ng/ml) and inactive (1.80 ± 1.86 ng/ml) acromegalic subgroups. However, significantly higher average hGH (7.00 ± 6.73 ng/ml *vs* 2.03 ± 2.86 ng/ml, *P* = 0.0180) and IGF-1 (501.3 ± 359.6 ng/ml *vs* 198.5 ± 79.1 ng/ml, *P* = 0.0060) concentrations were measured in active acromegalic subgroup compared to inactive one.

**Table 1:** Clinical data of acromegalic patients and age-matched control subjects.

	Control	Acromegaly
**Age (years)**	51.3 ± 7.6	55.7 ± 10.4
**Weight (kg)**	68.9 ± 14.7	87.7 ± 19.3**
**Height (cm)**	165.1 ± 10.5	168.9 ± 8.2
**BMI (kg m** ^**-2**^ **)**	25.1 ± 3.7	30.6 ± 5.3**
**Systolic BP (mmHg)**	126.9 ± 13.4	133.2 ± 17.7
**Diastolic BP (mmHg)**	75.5 ± 8.5	82.7 ± 12.4*
**0 min glucose (mmol l** ^**-1**^ **)**	5.04 ± 0.52	5.40 ± 0.71
**120 min glucose (mmol l** ^**-1**^ **)**	5.30 ± 1.30	6.30 ± 2.53
**HbA1c (%)**	5.70 ± 0.50	5.90 ± 0.74
**hGH nadir following OGTT (ng ml** ^**-1**^ **)**	1.02 ± 1.42	2.72 ± 2.13*
**IGF-1 (ng ml** ^**-1**^ **)**	151.0 ± 51.4	370.1 ± 311.8*
**IGF-1 x ULN**	0.50 ± 0.33 x ULN	1.66 ± 1.59 x ULN**

Abbreviations: BMI: body mass index; BP: blood pressure; HbA1c: glycosylated hemoglobin; hGH: human growth hormone; IGF-1: insulin-like growth factor-1; OGTT: oral glucose tolerance test; ULN: upper limit of normal value; n = 30 in each group,

*P<0.05,

**P<0.001 *vs* controls.

### Echocardiography measurements in study subjects

There were significant differences between the two groups in the echocardiographic dimensions. Patients with acromegaly exhibited significantly higher values in left ventricular end diastolic and end systolic diameter and in interventricular septum, left ventricular posterior wall thickness compared to age-matched controls (Table 2). These results were not unexpected and were supportive of the presence of myocardial hypertrophy of in the acromegalic patients and could be related to the duration and activity of the disease. However, no significant difference was detected in the echocardiographic parameters measured between active and inactive acromegaly subgroups (EF: 66.7 ± 7.4% *vs* 67.9 ± 6.4%, EDD: 52.6 ± 4.9 mm *vs* 52.7 ± 6.1 mm, ESD: 32.1 ± 5.9 mm *vs* 32.5 ±4.3 mm, IVS: 10.6 ± 1.3 mm *vs* 11.8 ± 3.0 mm, PW: 10.5 ± 1.3 mm *vs* 11.2 ± 1.6 mm, respectively).

**Table 2:** Echocardiographic parameters in patients with acromegaly and age-matched controls.

	Control	Acromegaly
**EF (%)**	70.6 ± 5.4	67.2 ± 6.9*
**EDD (mm)**	48.0 ± 3.9	52.6 ± 5.4*
**ESD (mm)**	29.1 ± 4.4	32.3 ± 5.2*
**IVS (mm)**	8.8 ± 0.7	11.1 ± 2.2**
**PW (mm)**	8.9 ± 0.7	10.8 ± 1.4**

Abbreviations: EF: ejection fraction; EDD: left ventricular end diastolic diameter; ESD: left ventricular end systolic diameter; IVS: interventricular septum; PW: posterior wall of left ventricle; n = 30 in each group

*P<0.05

**P<0.0001 *vs* controls

### Electrocardiographic parameters in study subjects

Comparison of the two groups (acromegalic patients *vs* control) revealed no significant differences in heart rate, the PQ, QRS and QT intervals and the QT dispersion. In order to reliably assess the duration of ventricular repolarization and to minimize the influence of changing heart rate on the QT interval, frequency correction of the QT interval (QTc) was performed by the Bazett, Fridericia, Framingham and Hodges formulas. QTc values calculated with all the four formulas showed no significant differences between acromegalic patients and controls. However, the T_peak_-T_end_ interval was significantly increased in acromegalic patients compared to controls (Table 3). Electrocardiographic parameters tended to be shorter in active acromegaly subgroup compared to the data measured in inactive subgroup (RR: 859.8 ± 134.5 ms *vs* 901.0 ± 178.2 ms, not significant (NS), QT: 392.7 ± 28.5 ms *vs* 412.0 ±29.5 ms, NS; QTc Bazett: 425.0 ± 16.0 ms *vs* 436.8 ±20.3 ms, NS; QTc Friderica: 413.7 ± 15.6 ms *vs* 428.1 ±16.6 ms, *P =* 0.0220; QTc Framingham: 414.3 ± 14.9 ms *vs* 427.3 ±17.8 ms, *P* = 0.0376; QTc Hodges: 412.5 ± 15.4 ms *vs* 426.9 ±16.7 ms, *P* = 0.0209; T_peak_—T_end_: 86.0 ± 15.7 ms *vs* 84.7 ±11.0 ms, NS, respectively).

**Table 3:** ECG parameters in patients with acromegaly and age-matched controls.

	Control	Acromegaly
**RR (ms)**	840.0 ± 75.0	877.6 ± 153.4
**PQ (ms)**	158.2 ± 17.7	158.0 ± 17.3
**QRS (ms)**	92.2 ± 6.5	95.3 ± 8.4
**QT (ms)**	389.3 ± 16.5	401.1 ± 30.0
**QTc (ms) Bazett**	425.6 ± 17.3	430.1 ± 18.6
**QTc (ms) Fridericia**	413.1 ± 14.5	419.9 ± 17.4
**QTc (ms) Framingham**	414.0 ± 13.7	419.9 ± 17.2
**QTc (ms) Hodges**	410.4 ± 13.8	418.7 ± 17.3*
**QTd (ms)**	36.6 ± 10.2	38.2 ± 13.2
**T**_**peak-**_ **T**_**end**_ **(ms)**	80.0 ± 10.3	85.5 ± 13.6
**STV**_**QT**_ **(ms)**	3.02 ± 0.80	4.23 ± 0.10**

Abbreviations: QTc: frequency corrected QT interval; QTd: QT dispersion; STV_QT_: short-term variability of QT interval; n = 30 in each group,

*P<0.05

**P<0.001 *vs* controls.

### Short-term beat-to-beat variability of the QT intervals

**Fig 1:**
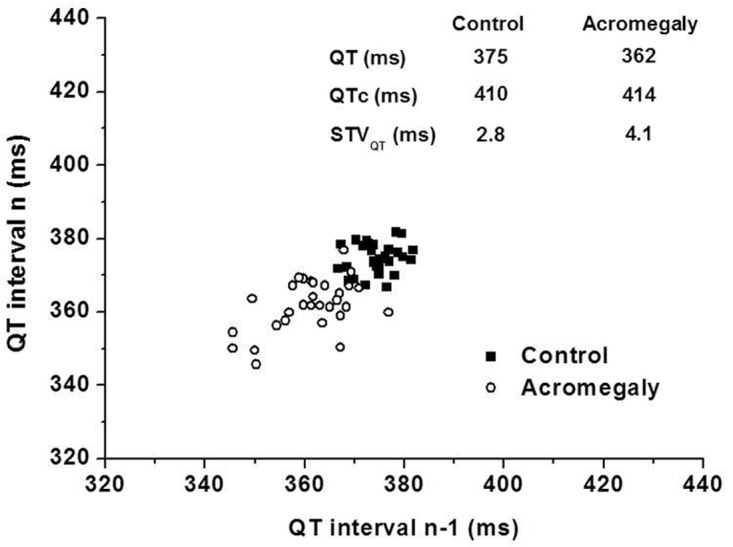
Representative Poincaré plots of a control individual and a patient with acromegaly. Note the larger area covered by data points obtained in the acromegalic patient illustrating increased short-term variability of the QT interval. Abbreviations: QTc, corrected QT interval by Bazett formula; STV_QT_, short-term variability of the QT interval.

**Fig 2:**
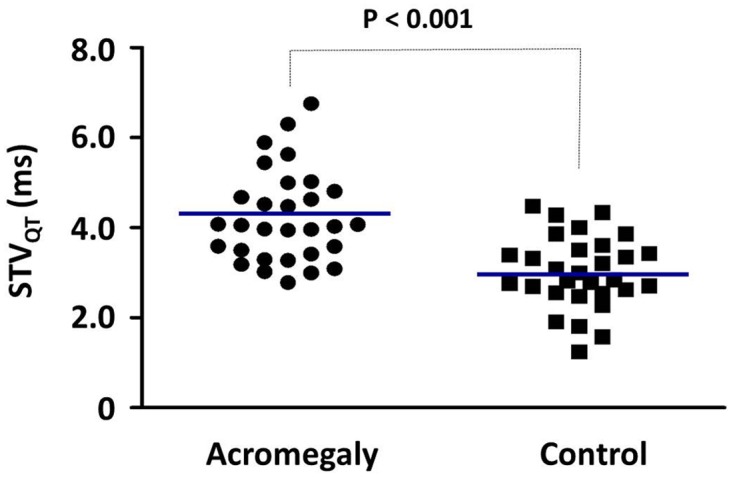
Short-term beat-to-beat temporal variability of the QT interval (STV_QT_) in acromegalic and matched control patients. Individual values measured in n = 30 patients in each group are presented, the blue lines indicate mean values, *P*<0.001.

To characterize the instability of cardiac ventricular repolarization, the short-term beat-to-beat variability of the QT interval was calculated in acromegalic patients and age-matched controls. As individual representative examples (Poincaré plots, Fig 1) and grouped average data show STV_QT_ was significantly increased by 36% in acromegalic patients compared to controls (4.23 ± 0.10 ms *vs* 3.12 ± 0.80, *P*<0.0001) (Fig 2). STV_QT_ values did not differ significantly between active (4.16 ± 0.89 ms) and inactive (4.33 ± 1.22 ms) acromegalic patient subgroups. There was no difference between acromegalic subjects treated with antihypertensive drugs (4.33 ± 0.95 ms, n = 18) and normotensive acromegalic patients (4.10 ± 1.16 ms, n = 12). We could not find any significant correlation between the STV_QT_ values and the left ventricular hypertrophy parameters in acromegaly patients or in the sub-groups of active and inactive patients (data not shown).

### Autonomic function

Standard cardiovascular reflex tests indicated significant deteriorations in Valsalva ratio (*P* = 0.0015), 30/15 ratio (*P* = 0.0143), and AN score (*P* = 0.0023) in patients with acromegaly, however, no significant differences in systolic blood pressure response after standing up, and diastolic blood pressure response after sustained handgrip were detected between the two groups (Table 4). AN score was significantly lower in active acromegaly subgroup, than in inactive group (2.1 ± 1.7 *vs* 3.9 ± 2.2; *P* = 0.0260), whereas other autonomic functions measured did not differ significantly in our two acromegalic subgroups (heart rate variation during deep breathing: 15.5 ± 6.5 min^-1^ *vs* 11.9 ± 7.95 min^-1^; Valsalva ratio: 1.50 ± 0.26 *vs* 1.40 ± 0.29; 30/15 ratio: 1.10 ± 0.13 *vs* 1.10 ± 0.29; Systolic blood pressure fall after standing up: 8.6 ± 11.8 mmHg *vs* 9.8 ± 6.2 mmHg; Diastolic blood pressure increase after handgrip: 19.1 ± 8.2 mmHg *vs* 15.4 ± 8.2 mmHg; respectively). There was no significant difference between the values of autonomic parameters measured in acromegalic subjects treated with antihypertensive drugs and normotensive patients with acromegaly.

**Table 4:** Autonomic neuropathy parameters of acromegalic patients and age-matched control subjects.

	Control	Acromegaly
**Heart rate variation during deep breathing (1/min)**	17.20 ± 6.44	14.00 ± 7.22
**Valsalva ratio**	1.70 ± 0.34	1.40 ± 0.28*
**30/15 ratio**	1.20 ± 0.18	1.10 ± 0.21*
**Systolic BP fall after standing up (mm Hg)**	6.40 ± 6.77	9.10 ± 9.66
**Diastolic BP increase after sustained handgrip (mm Hg)**	20.00 ± 9.22	17.50 ± 8.2
**AN score**	1.50 ± 1.28	2.90 ± 2.11*

Abbreviations: AN, autonomic neuropathy; BP, blood-pressure; 30/15 ratio, immediate heart rate response to standing; n = 30 in each group,

*P<0.05

**P<0.001 *vs* controls.

### Correlation of serum hGH and IGF-1 x ULN levels with cardiovascular data and autonomic neuropathy parameters

Pearson coefficient values indicated that neither hGH nor IGF-1 x ULN hormone level correlated with STV_QT_ or any other ECG parameters measured (Table 5). However, serum hGH concentration negatively correlated with diastolic blood pressure (*P* = 0.0326), thickness of posterior wall of left ventricle (*P* = 0.0333), and AN score (*P* = 0.0131), whereas IGF-1 x ULN levels positively correlated with Valsalva ratio (*P* = 0.0087).

**Table 5:** Correlation of serum average hGH and IGF-1 x ULN level of acromegalic patients with cardiovascular data and autonomic neuropathy parameters.

	Serum average hGH level (ng ml^-1^)		Serum IGF-1 x ULN level	
	Pearson r	*P value (two-tailed)*	Pearson r	*P value (two-tailed)*
**Systolic BP (mmHg)**	- 0.2294	*0*.*2313*	0.3206	*0*.*0900*
**Diastolic BP (mmHg)**	- 0.3978	*0*.*0326* *	0.1256	*0*.*5161*
**EF (%)**	0.3170	*0*.*0878*	-0.1735	*0*.*3593*
**EDD (mm)**	- 0.2911	*0*.*1186*	-0.1680	*0*.*3749*
**ESD (mm)**	- 0.3507	*0*.*0574*	-0.1076	*0*.*5716*
**IVS (mm)**	- 0.2714	*0*.*1469*	0.0388	*0*.*8387*
**PW (mm)**	- 0.3897	*0*.*0333* *	0.1048	*0*.*5815*
**RR (ms)**	- 0.1204	*0*.*5262*	- 0.1045	*0*.*5826*
**PQ (ms)**	- 0.1968	*0*.*2974*	- 0.0284	*0*.*8817*
**QRS (ms)**	- 0.0127	*0*.*9468*	- 0.3023	*0*.*1044*
**QT (ms)**	- 0.2032	*0*.*2815*	- 0.2360	*0*.*2092*
**QTc (ms) Bazett**	- 0.1090	*0*.*5663*	- 0.2084	*0*.*2690*
**QTc (ms) Fridericia**	- 0.1992	*0*.*2914*	- 0.2919	*0*.*1175*
**QTc (ms) Framingham**	- 0.1834	*0*.*3320*	- 0.2690	*0*.*1506*
**QTc (ms) Hodges**	- 0.2154	*0*.*2530*	- 0.2975	*0*.*1103*
**QTd (ms)**	0.1562	*0*.*4099*	0.1758	*0*.*3526*
**T**_**peak-**_ **T**_**end**_ **(ms)**	- 0.0917	*0*.*6298*	- 0.0788	*0*.*6791*
**STV**_**QT**_ **(ms)**	- 0.3401	*0*.*0659*	- 0.0924	*0*.*6272*
**Heart rate variation during deep breathing (1/min)**	0.2300	*0*.*2390*	- 0.1267	*0*.*5206*
**Valsalva ratio**	0.1340	*0*.*4967*	0.4864	*0*.*0087* *
**30/15 ratio**	0.2386	*0*.*2307*	0.0167	*0*.*9342*
**Systolic BP fall after standing up (mm Hg)**	- 0.3617	*0*.*0586*	- 0.2206	*0*.*2593*
**Diastolic BP increase after sustained handgrip (mm Hg)**	0.2421	*0*.*2146*	- 0.0762	*0*.*6998*
**AN score**	- 0.4714	*0*.*0131* *	- 0.2077	*0*.*2987*

Abbreviations: 30/15 ratio, immediate heart rate response to standing; AN, autonomic neuropathy; BP: blood pressure; EDD: left ventricular end diastolic diameter; EF: ejection fraction; ESD: left ventricular end systolic diameter; hGH: human growth hormone; IGF-1: insulin-like growth factor-1; ULN: upper limit of normal value; IVS: interventricular septum; PW: posterior wall of left ventricle; QTc: frequency corrected QT interval; QTd: QT dispersion; STV_QT_: short-term variability of QT interval; n = 30 for each number of XY pairs,

*P<0.05 for correlation.

## Discussion

Although a connection between acromegaly and increased cardiovascular morbidity and mortality has been established previously, this study is the first to demonstrate increased beat-to-beat short-term variability of the QT interval in acromegalic patients. There was no significant difference between STV_QT_ values measured in clinically and biochemically active acromegalic patients and those in inactive patients, which may suggest that elevated STV_QT_ is related to the presence of acromegaly and not to the efficacy of the treatments applied. STV_QT_ is a novel ECG parameter that, according to experimental [16–18] and clinical [20–23] data, more reliably predicts the development of serious ventricular arrhythmia compared to conventional ECG parameters of repolarization. STV_QT_ values may be used to help predict individual risk for arrhythmia and sudden cardiac death in patients with acromegaly, however, the efficacy of this approach could only be confirmed during prospective clinical studies.

Cardiac rhythm abnormalities have been demonstrated by electrocardiogram and Holter studies in acromegaly [4,5]. Resting electrocardiological changes included left axis deviation, increased QT intervals, septal Q-waves, ST-T wave depression, and late potentials in acromegalic patients [7,10]. Atrial and ventricular ectopic beats, paroxysmal atrial fibrillation, paroxysmal supraventricular tachycardia, sick sinus syndrome, bundle branch block, and ventricular tachycardia were seen during physical exercise [4,5]. The severity of ventricular arrhythmias correlated with increases in left ventricular mass and the frequency of ventricular premature complexes increased with the duration of acromegaly [8]. Fatti *et al*. [12] detected abnormally long QTc interval before treatment in one-quarter of 30 acromegalic patients in a retrospective study. Octreotide, a somatostatin analogue, was shown to reduce QT intervals [12], and reduce the number of ventricular premature complexes in acromegalic patients [26].

Acromegalic cardiomyopathy is frequently present at diagnosis and the majority of patients with acromegaly meet echocardiographic criteria for left ventricular hypertrophy [5]. A possible reason is that acromegalic patients are sometimes diagnosed only after longer duration (7–10 years) of the disease. No significant difference in left ventricle hypertrophy was observed between active and inactive acromegaly patients in our study, which may indicate that adequate treatment of acromegaly could not turn back the process. Cardiac performance of acromegalic patients during physical exercise depends on left ventricular diastolic function under resting condition [27]. Ciulla *et al*. [28] found elevated myocardial echoreflectivity and increased QTd in acromegalic patients and explained these changes by long-term, blood pressure-independent cardiac hypertrophy and prolonged exposure to high serum concentrations of hGH and IGF-1. Baykan *et al*. [29] analyzed echocardiographic parameters by tissue and two-dimensional Doppler imaging in acromegalic patients and found that GH level positively correlated with interventricular septum thickness. Our observations regarding these parameters were unexpectedly different. Myocardial hypertrophy in relevant animal models has been shown to result in electrophysiological remodeling where the expression of potassium channels critical for repolarization and repolarization reserve (such as I_Ks_), is significantly reduced, creating an arrhythmia substrate of increased spatial heterogeneity and temporal instability of repolarization and leading to increased arrhythmia susceptibility in the heart [14,30–32]. Patients with acromegaly may also develop congestive heart failure, the ratio was less than 3% (10 of 330 consecutive patients) in a study performed in 2 centers [33]. Recent studies indicated that I_Ks_, I_Kr_, I_K1_, and I_to_ potassium channels were down-regulated [34–36] and the persistent or slowly-inactivating sodium current was also increased in chronic heart failure [37]. Additionally, acromegalic patients could also develop coronary heart disease and most patients have systemic complications affecting the Framingham risk score [38]. GH receptor antagonist therapy improved the score and reduced the risk for coronary heart diseases [3]. In acromegalic patients, increased stiffness of ascending aorta was described [39] and ambulatory arterial stiffness indexes might have an important role in predicting cardiovascular risk [40]. Several mechanisms have been implicated in the development of ventricular arrhythmias in the settings of myocardial ischemia and myocardial infarction [41]. The surviving ventricular myocytes in the border zone next to the infarcted area play a particularly important role in the development of arrhythmias [42,43]. In these cells, a consistent downregulation of different potassium channels has been found, including I_to_ [42], I_K1_ [44], I_Kr_ and I_Ks_ [45]. The QT variability index, among the first ECG parameters used to characterize temporal variability of repolarization, has been shown to more reliably predict myocardial ischemia and myocardial infarction associated serious ventricular arrhythmia development compared to more conventional ECG parameters [15,46,47]. It should be noted that myocardial fibrosis occurring in acromegaly [48] can also contribute to the underlying arrhythmia substrate in the heart due to disturbances in conduction.

Animal studies support the cardiovascular findings of clinical observations on acromegalic patients. Overexpression of bovine GH gene increased cardiac mass, induced hypertrophy of left ventricle, and deteriorated cardiac systolic function in adult female transgenic mice [49]. The long-term exposure to high serum GH concentration also resulted in impaired high-energy phosphate metabolism and mitochondrial ultrastructural changes in the heart muscle of mice [49]. Bovine GH transgenic mice also developed a salt-resistant form of hypertension and structural narrowing of the resistance vasculature [50].

Our observations indicate deterioration in autonomic function assessed by standard cardiovascular reflex tests in acromegalic patients. AN score was significantly worse in inactive acromegalic patients and there was no apparent difference between acromegalic subgroups in other autonomic parameters measured, which may suggest that these neuropathy parameters are long-term consequences of acromegaly and cannot be reverted by the control of the disease. Among the tests primarily reflecting parasympathetic functions, the Valsalva ratio and 30/15 ratio were significantly decreased in acromegaly, whereas heart rate variation during deep breathing was not changed significantly. The tests demonstrating sympathetic activity, such as systolic blood pressure fall after standing up and diastolic blood pressure increase after sustained handgrip, did not change significantly in acromegalic patients. These reflex tests indicate a moderate parasympathetic dysfunction in our study, which could represent a predisposition to proarrhythmic activity in acromegalic patients. Increased risk of sudden cardiac death and ventricular arrhythmia has been associated with decreased parasympathetic and increased sympathetic activity [51]. Parasympathetic activation has been considered as antiarrhythmic regarding the development of ventricular fibrillation in pathological settings; for a recent review see [52]. There are conflicting data published about the cardiac autonomic functions in patients with acromegaly [53–57]. Dural *et al*. [53] provided evidence of sympathovagal imbalance due to sympathetic hypertone in acromegalic patients. Acromegaly was significantly associated with cardiac autonomic dysfunction independent from the presence of hypertension or diabetes mellitus [53]. Comunello *et al*. [54] analyzed 24 h frequency domain heart rate variability and found a correlation between reduced sympathovagal balance and pathological conditions, such as diabetes or hypertension in acromegalic patients. Chemla *et al*. [55] found that 10±6 months successful treatment of acromegaly could increase parasympathetic modulation and decrease sympathetic modulation of the night time heart variability and this effect was unrelated to changes in sleep apnea status. In contrast to our observations, sympathovagal imbalance due to increased vagal tone was demonstrated as a new risk factor for arrhythmias and syncope in acromegalic patients with left ventricle hypertrophy, although with normal heart rate, normal QT interval, and normal ejection function [56]. High frequency bands in orthostatism, but not in clinostatism, were higher in acromegalic patients than in normal subjects [56]. However, Seravalle *et al*. [57] have recently detected significantly decreased adrenergic tone through direct recording of muscle sympathetic nerve activity in newly diagnosed acromegalic patients with insulin resistance, but without cardiac hypertrophy.

Determination of beat-to-beat STV_QT_ is an intensively investigated new and non-invasive method for assessment of proarrhythmic risk [14,24]. QT interval measurements provide physiological information regarding the duration of cardiac repolarization. However, simple QT interval measurements are not always reliable in arrhythmic risk prediction. Ventricular repolarization is governed by a fine balance of inward and outward ionic currents. Under normal conditions impairment of one type of outward potassium channels is not likely to cause excessive QT prolongation, since other types of potassium channels provide sufficient repolarizing capacity. This was termed as repolarisation reserve [58,59]. Temporal STV_QT_ proved to be a more sensitive predictor of Torsades de Pointes ventricular tachycardia development than conventional QT parameters, such as QT and rate corrected QT intervals or the spatial QT interval dispersion, in case of experimentally impaired repolarization reserve [17,18,60]. There are numerous examples for the association between different pathophysiological conditions and attenuated repolarization reserve caused by electrophysiological remodelling. In addition, in experimental studies ventricular hypertrophy and chronic heart failure (CHF) were associated with decreased repolarisation reserve and/or high incidence of proarrhythmic events [17,61–63]. The significance and sensitivity of STV_QT_ as a predictor for electrical remodelling and proarrhythmia has recently been confirmed in clinical conditions in connection with CHF [22]. Increased STV_QT_ in the context of moderate CHF may reflect a latent repolarization disorder and increased susceptibility to sudden death in patients with dilated cardiomyopathy, which is not identified by a prolonged QT interval. In this study, increased STV_QT_ was the strongest indicator with an odds ratio of 1.52 (95% confidence interval 1.20 to 2.07, *P* = 0.007) for a history of documented ventricular tachycardia [22].

Varkevisser *et al*. [24] has recently reviewed the studies in which beat-to-beat STV_QT_ was a better indicator than QT interval prolongation for identification of healthy subjects or patients at risk for ventricular arrhythmias. In this regard, we have recently demonstrated that professional soccer players with hypertrophied hearts had increased STV_QT_ both in resting conditions and after exercise [64]. Significantly increased baseline STV_QT_ was able to identify patients with diminished repolarization reserve exhibiting drug-induced Torsades de Pointes [20] and those with inherited long QT syndrome [21]. Increase in STV_QT_ and prolongation of QT interval were observed in patients receiving cardiotoxic doxorubicin therapy [65]. A prospective clinical trial, the EUTrigTreat clinical study, was completed to investigate arrhythmogenic risk factors, including beat-to-beat variability of repolarization in sudden cardiac death risk stratification in patients with implantable cardioverter defibrillator [66]. Similar, prospective trials may elucidate the benefit of the use of STV_QT_ in other patient populations including acromegaly.

Increased temporal instability of cardiac repolarization characterized by elevated STV_QT_ in pathological situations, including acromegaly, can refer to impairment of repolarization reserve and increased propensity for arrhythmias [14]. In this setting, even relatively weak inhibition of potassium channels by seemingly harmless medications and/or dietary constituents may lead to sudden and unexpected excessive QT prolongation and development of Torsades de Pointes ventricular tachycardia [14].

Limitations of the study: It is important to note that in the present study the duration of acromegaly from the diagnosis can be defined (10–30 years), however the exact onset of the disease is not determinable and furthermore the duration since the remission in the inactive acromegalics is also not known. Therefore the real exposure time of increased hGH level before the diagnosis and effective treatment of the disease is not known and our active and inactive patients groups can be heterogenous in this regard. Moreover, the actual hormone levels used for correlation calculations with echocardiography and other cardiovascular parameters do not necessarily correspond to the duration of the disease. Because of our unexpected negative correlation between the GH level and the posterior wall thickness, further echocardiographical studies are warranted to examine the relationship between GH and IGF-1 levels and echocardiographic parameters in a larger series of acromegalic patients. A prospective study on newly diagnosed acromegaly patients could answer the question whether effective treatment would have any time-related effects on the changes in STV_QT_ variability and autonomic cardiovascular functions.

In conclusion, STV_QT_ is increased in patients with acromegaly while more conventional parameters of ventricular repolarization were unchanged. STV_QT_ values did not differ between active and inactive acromegalic patients and did not correlate with actual serum concentrations of hGH and IGF-1. These observations may suggest that elevated short-term beat-to-beat variability is a consequence of the disease and not related directly to current treatment or condition of the patient. The elevated STV_QT_ suggests instability of ventricular repolarization and may be an early indicator of increased liability to arrhythmia in patients with acromegaly. Further prospective clinical studies are needed to identify individual risk for ventricular arrhythmias in acromegalic patients.

## Supporting Information

S1 Table Clinical data of acromegalic patients. Abbreviations: BMI: body mass index; hGH: human growth hormone; IGF-1: insulin-like growth factor-1; OGTT: oral glucose tolerance test; ULN: upper limit of normal value.
(DOC)
