# Supplementary material for: Increased Short-Term Beat-To-Beat Variability of QT Interval in Patients with Acromegaly
Source: PLoS One. 2015 Apr 27;10(4):e0125639. doi: 10.1371/journal.pone.0125639 (PMC4411033; doi:10.1371/journal.pone.0125639)
Supplement: S1 Table — Abbreviations: BMI: body mass index; hGH: human growth hormone; IGF-1: insulin-like growth factor-1; OGTT: oral glucose tolerance test; ULN: upper limit of normal value. (DOC) [file pone.0125639.s001.doc]

**S1 Table. Clinical data of acromegalic patients**

| **Age (years)** | **Weight (kg)** | **Height (cm)** | **BMI**  **(kg m^-2^)** | **Serum average hGH (ng ml^-1^)** | **Serum IGF-1**  **(ng ml^-1^)** | **IGF-1 x ULN** | **Medical treatment of acromegaly** | **Acromegaly status** | **Antihypertensive**  **treatment** |
| --- | --- | --- | --- | --- | --- | --- | --- | --- | --- |
| 37 | 118 | 189 | 33,0 | 2.22 | 431 | 1.5 x ULN | Octreotide  (1x20 mg) | Active^a^ | - |
| 59 | 93 | 176 | 30.0 | 7.18 | 563 | 2.5 x ULN | no treatment | Active | Ramipril |
| 62 | 90 | 165 | 33.1 | 2.06 | 296 | 1.4 x ULN | no treatment | Active^a^ | Ramipril, amlopidin |
| 42 | 80 | 174 | 26.4 | 9.54 | 472 | 1.8 x ULN | Octreotide  (1x30 mg) | Active | - |
| 59 | 88 | 166 | 31.9 | 2.95 | 898 | 3.9 x ULN | Lanreotide  (1x120 mg) | Active | Metoprolol, amlopidin, indapamid |
| 76 | 82 | 163 | 30.9 | 1.64 | 334 | 1.9 x ULN | no treatment | Active | Enalapril, hydrochlorothiazide, doxazosin |
| 58 | 85 | 167 | 30.3 | 7.83 | 308 | 1.4 x ULN | no treatment | Active^a^ | Nebivolol |
| 55 | 84 | 160 | 32.8 | 10.46 | 662 | 2.8 x ULN | no treatment | Active | Enalapril, hydrochlorothiazide, amlodipin, bisoprolol, rilmenidin |
| 54 | 81 | 170 | 28.0 | 11.46 | 760 | 3.2 x ULN | no treatment | Active | - |
| 61 | 115 | 185 | 33.6 | 14.65 | 191 | 0.9 x ULN | no treatment | Active^a^ | Ramipril, hydrochlorothiazide, felodipin, metoprolol, doxazosin, rilmenidin |
| 72 | 74 | 170 | 25.6 | 1.70 | 1643 | 8.7 x ULN | no treatment | Active | Perindopril, amlodipin, indapamid |
| 34 | 94 | 184 | 27.8 | 3.17 | 355 | 1.2 x ULN | no treatment | Active^a^ | - |
| 43 | 68 | 170 | 23.5 | 28.30 | 151 | 0.6 x ULN | no treatment | Active^a^ | - |
| 62 | 70 | 169 | 24.5 | 5.50 | 378 | 1.8 x ULN | no treatment | Active | - |
| 57 | 77 | 155 | 32.0 | 3.41 | 473 | 2.1 x ULN | no treatment | Active | Perindopril, amlodipin, indapamid |
| 67 | 54 | 163 | 20.3 | 2.76 | 126 | 0.6 x ULN | no treatment | Active^a^ | Ramipril, felodipin |
| 61 | 71 | 164 | 26.4 | 4.91 | 481 | 2.3 x ULN | no treatment | Active | Perindopril, indapamid |
| 57 | 115 | 174 | 38.0 | 0.69 | 286 | 1.3 x ULN | no treatment | Inactive | Valsartan, metoprolol, doxazosin |
| 39 | 71 | 162 | 27.1 | 10.80 | 237 | 0.8 x ULN | Pegvisomant  (1x10 mg) | Inactive^b^ | - |
| 68 | 82 | 162 | 31.2 | 1.08 | 305 | 1.5 x ULN | no treatment | Inactive | Enalapril, amlodipin, doxazosin |
| 52 | 95 | 162 | 36.2 | 1.55 | 81 | 0.3 x ULN | no treatment | Inactive | - |
| 49 | 70 | 164 | 26.0 | 4.51 | 296 | 1.2 x ULN | Octreotide  (1x10 mg) | Inactive^b^ | - |
| 76 | 83 | 158 | 33.2 | 0.75 | 171 | 0.9 x ULN | Octreotide  (1x20 mg) | Inactive | Ramipril, metoprolol |
| 49 | 86 | 170 | 29.8 | 0.60 | 190 | 0.8 x ULN | no treatment | Inactive | Nebivolol, amlodipin |
| 49 | 78 | 173 | 26.1 | 1.70 | 146 | 0.6 x ULN | Bromocriptine  (4x5 mg) | Inactive | - |
| 56 | 101 | 168 | 35.8 | 1.97 | 292 | 1.3 x ULN | Bromocriptine  (2x5 mg) | Inactive | Valsartan, hydrochlorothiazide, amlodipin, carvediol, doxazosin |
| 54 | 92 | 168 | 32.6 | 1.15 | 147 | 0.6 x ULN | Bromocriptine  (2x5 mg) | Inactive | - |
| 54 | 155 | 182 | 46.8 | 0.05 | 75 | 0.3 x ULN | no treatment | Inactive | Ramipril, metoprolol, amlodipin, valsartan, prazosin |
| 52 | 80 | 172 | 27.0 | 0.99 | 186 | 0.8 x ULN | Bromocriptine  (1x2.5 mg) | Inactive | - |
| 58 | 100 | 162 | 38.1 | 0.53 | 168 | 0.8 x ULN | no treatment | Inactive | Ramipril, hydrochlorothiazide |

Abbreviations: BMI: body mass index; hGH: human growth hormone; IGF-1: insulin-like growth factor-1; OGTT: oral glucose tolerance test; ULN: upper limit of normal value

^a^ active patient with normal IGF-1 x ULN, but high serum average hGH level

*Note:* Active acromegaly was defined as high serum average hGH and/or high IGF-1 x ULN. „Normal” hGH is defined as 0-8 ng ml^-1^ for healthy adults and for controlled disease as random hGH < 1 ng ml^-1^ according the new guideline. Previously it was 2.5 ng ml^-1^. „Average” hGH was measured according to the guideline by the assessment of hGH burden: 5-point day curve for GH. The IGF-1 values are age-sex adapted in normal range and high IGF-1 was defined as > 1.5 x ULN.

^b^ inactive patient with high serum average hGH level and normal IGF-1 x ULN

*Note:* Controlled or inactive acromegaly is defined as an age-sex-appropriate normal IGF-1 and/or random GH < 1 ng ml^-1^ and/or nadir GH after OGTT < 0.4 ng ml^-1^, but is almost good around or below 2.5 ng ml^-1^. The reason of the high serum average hGH level is that one „inactive” acromegalic patient received GH receptor antagonist Pegvisomant treatment, so the GH average level of this patient was obviously high, however, the IGF-1 level was low as the consequence of the treatment. The other patient is on 10 mg Octreotide therapy, IGF-1 is normal, higher average hGH means that the dose of Octreotide should be increased by 1x20 mg.
